# Resistance of *Argopecten purpuratus* scallop larvae to vibriosis is associated with the front-loading of immune genes and enhanced antimicrobial response

**DOI:** 10.3389/fimmu.2023.1150280

**Published:** 2023-03-03

**Authors:** Eduardo Jeria, Daniel Oyanedel, Rodrigo Rojas, Rodolfo Farlora, German Lira, Ana Mercado, Katherine Muñoz, Delphine Destoumieux-Garzón, Katherina Brokordt, Paulina Schmitt

**Affiliations:** ^1^ Laboratorio de Genética e Inmunología Molecular, Instituto de Biología, Pontificia Universidad Católica de Valparaíso, Valparaíso, Chile; ^2^ Laboratorio de Patobiología Acuática, Departamento de Acuicultura, Universidad Católica del Norte, Coquimbo, Chile; ^3^ Laboratorio de Biotecnología Acuática y Genómica Reproductiva (LABYGER), Instituto de Biología, Facultad de Ciencias, Universidad de Valparaíso, Valparaíso, Chile; ^4^ Centro de Investigación y Gestión de Recursos Naturales (CIGREN), Universidad de Valparaíso, Valparaíso, Chile; ^5^ Laboratorio de Fisiología y Genética Marina (FIGEMA), Departamento de Acuicultura, Universidad Católica del Norte, Coquimbo, Chile; ^6^ IHPE, Université de Montpellier, CNRS, Ifremer, Université de Perpignan Via Domitia, Montpellier, France

**Keywords:** mollusk, scallop-*Vibrio* interaction, invertebrate immunity, immune front-loading, resistance to diseases

## Abstract

Mass mortality events caused by vibriosis have emerged in hatchery-reared scallop larvae from Chile, threatening scallop aquaculture. In an attempt to mitigate this emerging infectious disease and provide candidates for marker-assisted selective breeding, we tested here the existence of a genetic component of *Argopecten purpuratus* scallop resistance to the pathogen *Vibrio bivalvicida.* Through a dual RNA-seq approach we analyzed the basal transcriptome and the transcriptional response to infection in two resistant and two susceptible families as well as the pathogen transcriptomic response to host colonization. The results highlighted a genetic basis in the resistance of scallop larvae to the pathogen. The *Vibrio* response was characterized by a general metabolic adaptation to the host environment, along with several predicted virulence factors overexpressed in infected scallop larvae with no difference between resistant and susceptible host phenotypes. On the host side, several biological processes were enriched in uninfected resistant larvae. Within these enriched categories, immune-related processes were overexpressed, while morphogenesis, biomineral tissue development, and angiogenesis were under expressed. Particularly, genes involved in immune recognition and antimicrobial response, such as lipopolysaccharide-binding proteins (LBPs), lysozyme, and bactericidal permeability-increasing protein (BPI) were overexpressed in uninfected resistant larvae. As expected, immune-related biological processes were enriched in *Vibrio-*infected larvae, but they were more numerous in resistant larvae. Overexpressed immune genes in response to infection included several Toll-like receptors, TNF and NF-κB immune signaling genes, and the antimicrobial peptide Big defensin ApBD1. Results strongly suggest that both a front-loading of immune genes and an enhanced antimicrobial response to infection contribute to the resistance, while pathogen infective strategy does not discriminate between host phenotypes. Overall, early expression of host immune genes appears as a strong determinant of the disease outcome that could be used in marker-assisted selective breeding.

## 1 Introduction

As filter-feeding organisms, scallops are continuously exposed to a large number of microorganisms present in the surrounding aquatic environment, and several infectious agents, such as bacteria, have been described (1). Consequently, the occurrence of mass mortalities of bivalves worldwide in recent decades has affected scallop production (2). In the case of *Argopecten purpuratus*, one of the bottlenecks preventing the expansion of the activity is the occurrence of mass mortality events in larvae produced in hatcheries, which have been associated with bacterial pathogens from the genus *Vibrio*, such as several strains of *V. splendidus* (2) and *V. bivalvicida* (2–4). Infection is initiated by the entry of the bacteria into the larva during the filter-feeding process. Then, *Vibrio* sequentially colonize the larval intestine and later spread to the other organs (4). Clinical symptoms of vibriosis include erratic swimming, veil disruption, and detachment of ciliated cells in the veil, which eventually leads to scallop larval death (4). So far, the only measure adopted to control these outbreaks is the use of antibiotics, which goes against the current trend of reducing this practice in aquaculture due to the dramatic increase in antibiotic resistance in bacteria (5).

Mass mortalities of bivalves worldwide have motivated the study of molluscan immune response due to the key role of host immunity in the defense against pathogens. As a result, characterization of the genes and immune mechanisms underlying the defense of scallops has increased in recent years, including *A. purpuratus* (6–11). However, almost all studies have focused on adult organisms, leaving the immune defense mechanisms of larvae poorly characterized, when this is the stage that suffers the disease. The few studies available on scallop larval immunity reflect that the ontogeny of the immune system begins early in development and that mature larvae can express immune-related genes in response to bacterial challenge (12).

Improving production efficiency and aquaculture success can be achieved by applying various types of genetic management strategies, such as selective breeding programs (13). Selective breeding has been effectively developed in marine bivalves, indicating a strong genetic basis and a positive response to selection that increases survival against various pathogens (14–16). Recently, it was shown that groups of juvenile oysters with contrasting resistance phenotypes to the Pacific oyster mortality syndrome (POMS) exhibit significant variations in the expression of immune response genes (17). Specifically, an elevated basal expression levels of genes associated with pathogen recognition proteins and immune signaling pathways were observed in oysters resistant to mortalities (17).

The outcome of infectious diseases depends on the capacity of hosts to control pathogens and on the strategies that pathogens have evolved to overcome the host immunity (1). This knowledge is required to design appropriate mitigation approaches in aquaculture. Currently, we lack a comprehensive view of (i) the genetic component associated to the resistant of scallop larvae to *V. bivalvicida*, (ii) the scallop factors that play a role facing *Vibrio bivalvicida* infection, and (ii) the molecular mechanisms underpinning the infectious process of *Vibrio bivalvicida* in *A. purpuratus* scallop larvae.

In this study, we produced larvae from scallop families and found that they exhibited contrasting resistance to the pathogen *Vibrio bivalvicida*. We characterized by dual RNA-seq both the pathogen transcripts related to the infectious process and the scallop transcripts associated with resistance. The *Vibrio* response was characterized by its adaptation to the host environment and expression of virulence factors independently of the scallop phenotype (resistant or susceptible). In scallop larvae, the frontloaded expression of host immune effector genes and expression of genes involved in recognition and in cellular and humoral antimicrobial mechanisms were identified as key determinants of the disease outcome. This novel approach is proposed as a new tool to elucidate the basis of the resistance phenotype that could be used in marker-assisted selective breeding.

## 2 Materials and methods

### 2.1 Production of larvae of *A. purpuratus*

All experiments that included scallops complied with regulations on the protection of animals used for experimental and scientific purposes (18).

Scallop larvae were produced in the central marine culture laboratory of the Universidad Católica del Norte (LCCM-UCN). To determine the *Vibrio bivalvicida* (VPAP30) dose to obtain 50% affected larvae, multiparental larvae were obtained from induced spawning as described in (19). Briefly, 80 mature adults (7.0 ± 0.5 cm in height) of *A. purpuratus* were collected from a culture in Tongoy Bay (Coquimbo, Chile). The organisms were maintained in a 1000 L aquarium with filtered seawater (1 µm) for two days and then spawning was induced by exposing mature scallops to a high concentration of microalgae (*Isochrysis galbana* clone T-iso + *Chaetoceros calcitrans* + *Pavlova lutheri*, 17 x 10^6^ cells/mL). When spawning was initiated, adults were separated to collect male and female gametes separately to avoid self-fertilization. Gamete products (oocytes from 65 individuals and sperm from 15 others) were mixed at a ratio of 7-10 sperm per oocyte, and the resulting eggs (~120×10^6^) were kept in a 250 L cylindrical tank filled with filtered (1 µm) and sterilized (UV) seawater, maintained at room temperature (17 ± 1°C) and with continuous aeration, for 21 days. Larvae were maintained in these tanks at a density of 15-25 larvae per mL (range used in scallop larval hatcheries). Larvae 48 h post-fertilization (hpf) were fed daily ensuring that a concentration of 20,000 cells-mL^-1^ was maintained in the tank. Feed rations were adjusted to larval density every other day.

To obtain scallop larvae of biparental origin, the nested crossing and conditioning design of animals was employed following a previously established protocol (20). Briefly, twenty-one full-sibling larval families were produced using sexually mature *A. purpuratus* adults (7 cm in length and 16 months of age) from a culture at Tongoy Bay, Chile. Each scallop was induced to spawn separately by excess microalgae as described above. The crosses were performed following a nested paternal half-sibling design in which gametes from one male were used to fertilize oocytes from three females chosen at random from the base population, for a total of 7 males and 21 females. Post-hatching, larvae from each full-sibling (FS) family were allowed to grow for 2 days and obtained D larvae were transferred to 250 L tanks (one tank per FS family). Larvae were maintained as described for larvae of multiparental origin for 21 days, until they reached the 200 µm size that corresponds to the late veliger larval stage.

### 2.2 Determination of the dose of the pathogen affecting 50% larvae (LD50/mL)

A culture of *V. bivalvicida* VPAP30 was grown on trypticase soy agar (TSA) medium supplemented with 2% NaCl at 22°C for 24 h. The culture was subsequently centrifuged 8000 x g for 5 min at 22°C and two washes were performed with sterile seawater (SSW). The concentration of colony-forming units (cfu)/mL was adjusted by measuring the optical density at 600 nm and dilution in SSW. The number of cfu/mL was verified by serial dilutions and plating in Trypticase-soy agar with 2% NaCl. Determination of the dose of the pathogen that affected 50% larvae was performed on the late veliger larvae of multiparental origin that were washed with SSW and sieved to remove organic matter. Next, 100 larvae/well were placed in a final volume of 4 mL of SSW in 6-well plates with gentle aeration. The VPAP30 strain was then added and tested at different concentrations (1x10^6^, 1x10^5^, 1x10^4^, 1x10^3^, and 1x10^2^ cfu/mL). As a control, 6 wells with pathogen-free larvae were included. Larvae were incubated at 18°C with gentle aeration achieved through 38G needles pierced through the lead of each well and connected to an air blower. After 24 h exposure the larval status was assessed as previously described (4), using an Olympus CKX41 inverted optical microscope (200X). Clinical signs of infected larvae, such as no ciliary movement, erratic swimming, larval crowding, veil shedding, and bacterial swarming around larvae were assessed as described in (4). Larvae that showed at least one of these clinical signs were considered as affected by the pathogen. After visual analysis, larvae were stained with lugol to determine the total number of larvae and the percentage of affected larvae. The percentage of affected larvae was calculated using the formula: % affected larvae =100* (Number of affected larvae)/(Number of total larvae).

### 2.3 Identification of biparental families of larvae with contrasting susceptibility to *V. bivalvicida*

Each of the 21 families produced in this study was infected with the dose of VPAP30 that affected 50% multiparental scallop larvae after 24 h. All larval families were evaluated by visual inspection prior to infection, and only families showing healthy larvae with normal swimming behavior and ciliary movements were analyzed. Time 0 h (uninfected) and 8 h (post-infection) were considered for larval collection for total RNA extraction and transcriptomic analysis, and time 24 h for the determination of the effect of VPAP30 on the larvae of each family. The experimental units consisted of glass flasks containing 1 L of SSW with 15 larvae/mL for sample collection for total RNA extraction, kept in a thermoregulated bath at 17°C (rearing temperature) and with aeration; or 3 replicates of 100 larvae/well in a final volume of 4 mL of SSW in 6-well plates with aeration to evaluate the effect of VPAP30 at 24 h as explained above (clinical signs). Control groups in triplicate of uninfected larvae from each family were included. For each family, experiments were conducted once, including 3 independent experimental units for each experimental condition, collecting 3 replicates of 15,000 larvae before infection and 3 replicates at 8 h post-infection.

### 2.4 Total RNA extraction from larvae and construction of cDNA libraries

To simultaneously capture pathogen and host transcripts, high-quality total RNA was isolated from scallop larvae using TRIzol^®^ reagent according to the manufacturer’s instructions (Thermo Scientific). To obtain the outside-host transcriptome from the pathogen, high-quality total RNA from *Vibrio bivalvicida* VPAP30 culture maintained in seawater for 8 h and concentrated by centrifugation was isolated as described above. Total RNA from scallop larvae and the pathogen was then treated with DNAse I (Thermo Scientific), for 15 min at room temperature and heat-inactivated for 10 min at 65°C, followed by a second precipitation with 0.3M sodium acetate (pH 5.2) and isopropanol (1:1 V:V). RNA concentration and purity were checked using a Nanodrop spectrophotometer (Thermo Scientific), and their integrity was analyzed by capillary electrophoresis on a BioAnalyzer 2100 (Agilent). Finally, 24 cDNA libraries from RNA samples from 4 larval families under uninfected and infected conditions for 8 h and from *Vibrio bivalvicida* VPAP30 were prepared using the TruSeq RNA Sample Preparation Kit v2 (Illumina^®^) by Novogene USA.

### 2.5 Sequencing of cDNA libraries and bioinformatics analysis

The cDNA libraries were sequenced by the NovaSeq 6000 platform (Illumina^®^) using sequencing runs of 2×150 paired-end reads by Novogene, USA. All the raw reads were submitted to the sequence reads archive (SRA), NCBI database with an accession number of PRJNA891447. After removal of adapters from the obtained sequences and low-quality sequences, the cleaned sequences were mapped against the *A. purpuratus* draft genome (available at NCBI, accession PRJNA418203) to filter out sequences that did not correspond to the species. Because the available genome corresponds to a draft genome, the previously annotated *de novo* assembled transcriptome (8) was used as a reference for differential gene expression (DEGs) analysis by RNA-seq. The clean sequences obtained from each library were mapped against the reference transcriptome (consisting of 48,076 contigs) using CLC Genomics Workbench software (Version 11.0.1, CLC Bio, Denmark). The difference between the rate of reads mapped against the reference genome and the rate of reads mapped against the reference transcriptome was around 20% and it was considered as missing data. The RNA-seq settings used were a minimum length fraction of 0.8 and a minimum similarity fraction (long reads) of 0.9. Expression values were set as transcripts per million mapped reads (TPM). To identify differential gene expression between experimental conditions and times analyzed, the differential expression tool for RNA-seq included in the CLC Genomics Workbench software was used. For the specific panel of immune genes, differentially expressed genes (|log_2_ Fold Change (FC)| ≥ 2; false discovery rate (FDR) p-value < 0.05 using T-test) were determined and visualized in a hierarchical clustering heat map, with Euclidean distance and full linkage set as parameters for analysis.

Reads that did not map to the scallop genome (**Supplementary Table 1**) were mapped to the *V. bivalvicida* VPAP30 genome (GenBank GCA_001188205.1) and used as reference for differential gene expression (DEGs) analysis by RNA-seq. To annotate the *Vibrio* DEGs between the outside-host and inside-host into functional categories, we recover the Gene Ontology (GO) data available for the transcriptome of *V. bivalvicida* VPAP30 using the ID mapping tool of the UniProt database (https://www.uniprot.org/id-mapping). DEGs not assigned to a biological process were assigned to a molecular function and those with no assigned molecular function were assigned to a cellular component. We were able to functionally annotate 1085 out of 1397 DEGs to different GO categories of biological function, molecular function, and cellular compartment. To focus on the virulence function, we used the VFanalizer pipeline (http://www.mgc.ac.cn/cgi-bin/VFs/v5/main.cgi?func=VFanalyzer) (21) to predict virulence factors (VFs) present in the genome of *V. bivalvicida* VPAP30 and to identify VFs DEGs between the outside and inside-host environment.

For the scallop transcriptome, functional enrichment analysis by GO was performed using the Rank-based Gene Ontology Analysis with Adaptive Clustering (RBGOA) tool (22), which allows to identify enriched biological processes and determine if they are overexpressed or downregulated according to the log_2_FC values of the DEGs contained on each process. We considered for the analysis 17,880 transcripts that showed an E-value less than 0.01 with sequences from the UniProt database (37.19% of the total contigs from the *de novo* scallop transcriptome). For the RGBOA analysis, the log_2_FC value was assigned to genes with an FDR equal or lower than 0.05 (significant) and a 0 to those with an FDR higher than 0.05 (non-significant) to include not only the significance of the differential expression of each gene but also the strength of the expression.

### 2.6 Validation of differential gene expression by RT-qPCR

The same RNA used for the construction of cDNA libraries was used for RT-qPCR amplification. The cDNA was synthesized from 1 µg RNA, using an AffinityScript qPCR cDNA synthesis kit (Stratagene) according to the manufacturer’s instructions. The primer pairs are listed in **Supplementary Table 2**. RT-qPCR assays were performed in duplicate on a Biorad C1000 Touch CFX96 thermal cycler using the Takyon ROX SYBR 2X qPCR kit (Eurogentec), and the efficiencies (E) of the primer pairs were calculated from six serial dilutions of pooled cDNA for each primer pair according to the equation E = 10^[-1/slope]^. Only primers with E between 95% and 105% were considered. The expression of candidate genes and reference genes was measured in 1 µL of 1:5 diluted cDNA. Assays were subjected to an initial denaturation step of 3 min at 95°C, followed by cDNA amplification (40 cycles of denaturation at 95°C for 10 s, alignment at 57°C for 10 s, and extension time at 72°C for 15 s) and fluorescence detection, and a final melting curve detection. Relative expression was calculated by the -2^DDCq^ method (23) using the geometric mean of the measured quantitation cycle (Cq) values of the constitutively expressed genes elongation factor 1 α (EF-1α) (24), and the enzyme Glyceraldehyde 3-phosphate dehydrogenase GAPDH to normalize the measured Cq values of the target genes.

### 2.7 Statistical analysis

Principal component analysis (PCA) was performed on the transcripts per million (log TPM) values of the cDNA library samples and plotted in the two-dimensional space spanned by the first and second principal component of the covariance matrix with 95% confidence ellipses using the FactoMineR package (25). The statistical difference of transcript expression was obtained from RNA-seq data (TPM values) based on the selection criterion on a P value FDR < 0.05. Calculations of means, standard deviations, and statistical analysis using the Kruskal-Wallis test for qPCR expression analysis were performed with GraphPad Prism software version 8.01 (significant value: *P* < 0.05). To compare the differential expression of the immune genes the hierarchical cluster analysis with p-values using the pvclust package was performed in R studio (26).

## 3 Results

### 3.1 Resistance of scallop larvae to *Vibrio bivalvicida* has a genetic basis

To identify families of larvae with potential contrasting resistance to the pathogen, we first used scallop larvae from multiparental origin to determine the *V. bivalvicida* VPAP30 dose to obtain 50% affected larvae (**Supplementary Table 3**; **Supplementary Figure 1**). Larvae were tested at 21 days post-fecundation, which corresponds to the late veliger stage (180-200 μm). After a 24 h exposure to ~1x10^5^ cfu/mL *V. bivalvicida*, 50% larvae showed the classical signs of vibriosis, such as erratic swimming, veil disruption and detachment of ciliated cells in the veil (4). This *Vibrio* dose was next used to determine the resistance of 21 biparental families of scallop larvae to the pathogen. Late veliger larvae were produced by a nested paternal half-sibling design (see material and methods).

**Figure 1:**
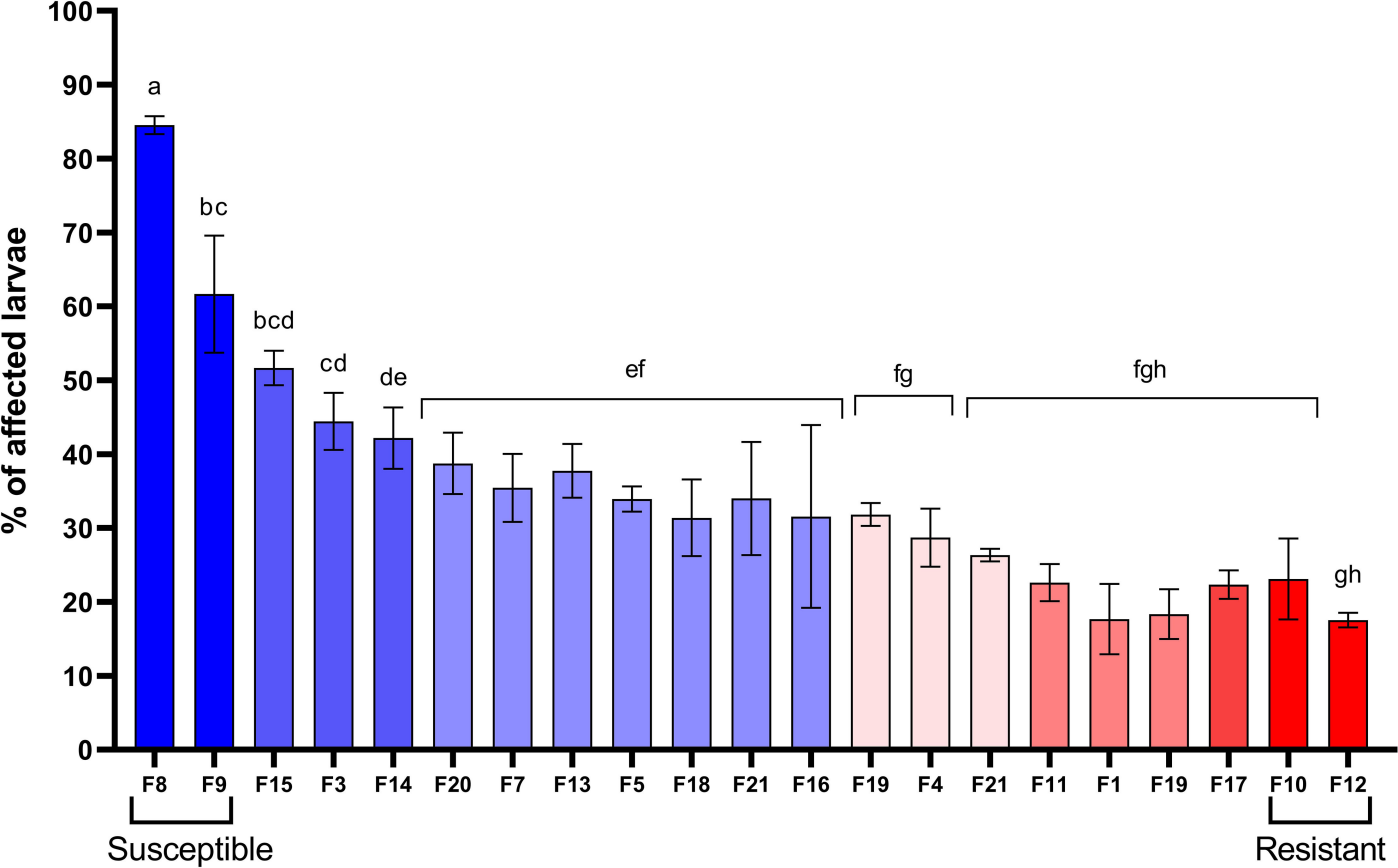
Identification of biparental families of *Argopecten purpuratus* scallop larvae with contrasting susceptibility to *Vibrio bivalvicida*. Scallop larvae (late veliger) from 21 biparental families (F) were infected with *V. bivalvicida* VPAP30 (1 x10^5^ cfu/mL) for 24 h before determination of affected larvae. Assays were performed in triplicate. The four scallop families used for transcriptomic analyses were highlighted as Resistant (F10, F12) or Susceptible (F8, F9). Different lowercase letters inside the graph indicate significant differences in percentage of affected larvae among scallop families (*P* < 0.05).

Biparental families of scallop larvae showed significant variability in the resistance to *V. bivalvicida*. Affected larvae ranged from 17.5% to 84.5% depending on the scallop family (**Figure 1**). Families F12 and F10 showed the highest resistance with only 17.5 ± 1.6% and 23.1± 9.4% affected larvae, respectively. By contrast, families F8 and F9 showed the highest susceptibility with 84.5 ± 2.1% and 61.6 ± 13.7% affected larvae, respectively. The other 17 families showed variable resistance between the most contrasted phenotypes. Interestingly, F10 and F12 were half-siblings, and also were F8 and F9. The four scallop families that showed the most contrasting phenotypes in the resistance to VPAP30 were used for transcriptomic analyses.

### 3.2 Early response of *V. bivalvicida* to the inside-host environment is independent of the host resistance

**Figure 2:**
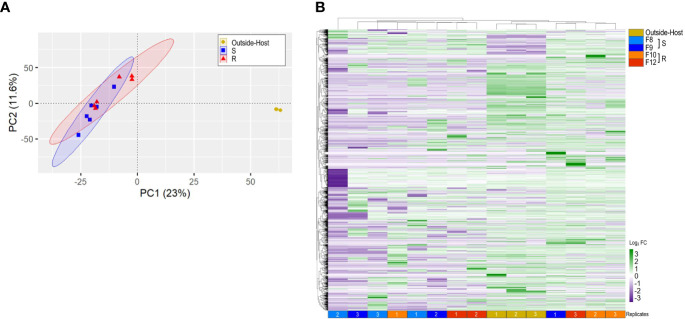
*Vibrio bivalvicida* VPAP30 inside-host and outside-host transcriptome analysis. **(A)**. Principal component analysis (PCA). Six cDNA libraries per host phenotype (resistant or susceptible) were included in the analysis. Yellow: outside-host; blue: Susceptible inside-host (S); red: Resistant inside-host (R). **(B)**. Hierarchical clustering of differentially expressed genes (DEGs) from *Vibrio bivalvicida* outside-host (yellow) and inside-host transcriptomes. Light blue and blue: Susceptible inside-host (S); orange and red: Resistant inside-host (R). cDNA library replicates are indicated at the bottom of the heat map. DEGs values are represented through the color scale from purple (relative low level of gene expression) to green (relative high level of gene expression).

To identify *Vibrio* transcripts modulated during the infectious process, we compared the inside-host and outside-host transcriptomes of the bacteria. To this end, we sequenced the transcriptome of *V. bivalvicida* in contact for 8 h with the four scallop families (inside-host) and after 8 h in seawater (outside-host, control). Between ∼20,283 and 217,151 reads mapped to the *V. bivalvicida* genome after filtering the reads that mapped to the host genome. This corresponds to an average of 5.3% of total mapped reads (**Supplementary Table 1**), which agrees with values found in previous dual RNA-seq analyses (27, 28). PCA analysis (**Figure 2A**) and hierarchical clustering (**Figure 2B**) clearly separated the inside-host and outside-host transcriptomes (gene expression profile), representing a major reprogramming of *Vibrio* transcriptome to the intra-host environment. However, similar *Vibrio* gene expression profiles were observed in susceptible and resistant larvae.

**Figure 3:**
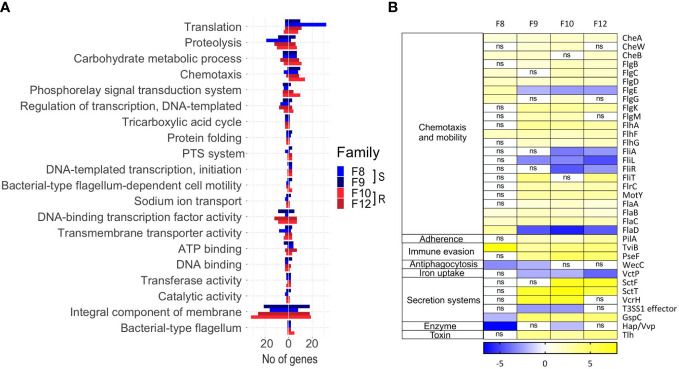
Inside-host *Vibrio bivalvicida* VPAP30 transcriptomes (8 hours after infection in *Argopecten purpuratus* larval families with contrasting vibrio-resistance phenotypes) and outside-host transcriptomes (control). **(A)**. The number of under or overexpressed DEGs (FDR < 0.05) belonging to different GO categories are displayed. DEGs with no GO annotation are not included. **(B)**. Virulence-centered annotation of the within-host bacterial DEGs. The heatmap shows the log_2_ fold-change of predicted virulence factor belonging to different functional categories associated with pathogenicity.

In order to unravel the general strategy used by *V. bivalvicida* to infect scallop larvae, we functionally annotated the *Vibrio* genes differentially expressed during colonization in each scallop family. Twenty out of 80 functional categories were common to all larval families (**Figure 3A**; **Supplementary Table 4**). Major metabolic and behavioral changes of *Vibrio* were observed. Most modulated functions included translation, carbohydrate utilization and chemotaxis, suggesting changes in *Vibrio* metabolic activity and mobility inside scallop larvae (**Figure 3A**). In agreement, functional annotation analysis of *Vibrio* transcripts indicated highly active global metabolism ranging from gene expression (initiation and regulation of transcription) to the synthesis and post-translational modifications of proteins.

Functional annotation based on GO terms provided a general landscape of the inside-host metabolic changes of *V. bivalvicida.* We next searched specifically for virulence factors being modulated during the colonization of the inside-host environment. Potential virulence factors (VF) were identified in the *Vibrio* genome (**Supplementary Table 5 tab 1**) and we focused on those differentially expressed after contact with scallop larvae (**Figure 3B**). Virulence factors classes involved chemotaxis and motility, adherence, immune evasion, antiphagocytosis, iron uptake, secretion systems, enzyme, and toxins (**Figure 3B**). *Vibrio* VFs expression was variable among scallop families, but not associated with the resistance phenotype. The most highly modulated genes were components from a type-III secretion system (log_2_FC 6.14, 7.95), as well as genes from the a type II secretion system (log_2_FC -1.9, 4.74); chemotaxis genes (*cheA, cheW, cheB*) (log_2_FC 0.93, 1.74), flagellar motility genes (*Flh, Flg, Fli, Fla* and *Flr* genes) (log_2_FC -6.04, 3.0), and the UDP-N-acetylglucosamine (UDP-GlcNAc) C-6 Dehydrogenase (*tviB*) (log_2_FC 3.11, 7.67) involved in VI capsular polysaccharide synthesis (**Figure 3B**).

### 3.3 Susceptible and resistant scallop larvae display differential transcriptomic profiles

**Figure 4:**
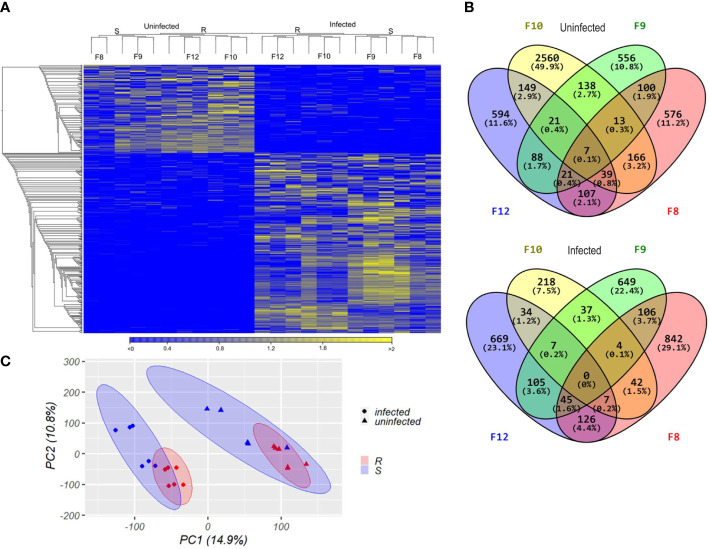
Transcriptomic analyses from susceptible and resistant *Argopecten purpuratus* scallop larvae, uninfected or infected with *Vibrio bivalvicida* VPAP30 for 8 h **(A)**. Hierarchical clustering of differentially expressed genes. cDNA library replicates clustered for susceptible (S, F8 and F9) or resistant (R, F10 and F12) larvae. DEGs were determined with a minimum |log_2_ fold change| >2; false discovery rate (FDR) p-value < 0.05 and expression values are represented through the color scale from blue (relative low level of gene expression) to yellow (relative high level of gene expression). On top, the dendrogram shows the relation among sample replicates. **(B)**. Principal component analysis (PCA) from the transcripts per million (TPM) values of the cDNA library samples which were plotted in the two-dimensional space spanned by the first and second principal component of the covariance matrix. Dots: infected larvae. Triangles, uninfected larvae. In red, resistant scallop larvae; in blue, susceptible scallop larvae. Confidence intervals for each host phenotype are indicated with colored ellipses. **(C)**. Venn diagrams of unique and common transcripts among the transcriptomes from uninfected and infected scallop larval families, showing the number and percentage of all classified transcripts included for each category.

Next, we analyzed the transcriptomes of the four scallop families. Transcriptomes were compared according to scallop phenotypes (resistant or susceptible) in both an uninfected (0 hpi) and a *Vibrio-*infected (8 hpi) state. RNA sequencing yielded between 71,560,270 and 130,983,504 individual Illumina reads per sample, 84.90% to 98.78% of which mapped to the scallop genome (**Supplementary Table 1**). We observed strong differential gene expression between uninfected and infected larvae by hierarchical clustering and PCA analysis ((**Figures 4A, B**), revealing that all scallop families responded to *Vibrio* exposure with major reprograming of the transcriptome after infection. Furthermore, host transcriptomes clearly clustered according to resistance phenotype (resistant or susceptible) and infection condition (uninfected or infected) (**Figures 4A, B**).

We analyzed the differentially expressed genes (DEGs) found between resistant and susceptible larvae before (0 hpi) and after (8 hpi) infection (**Supplementary Table 3**; **Supplementary Figures 2A, B**). Before infection, a total 349 genes were differentially expressed between resistant (173 DEGs) and susceptible (176 DEGs) larvae. After infection, a total 378 genes were differentially expressed between resistant (180 DEGs) and susceptible (198 DEGs) larvae. Independently of the infection status, 101 DEGs were specific to resistant scallops whereas 110 DEGs were specific to susceptible scallops (**Supplementary Table 3**; **Supplementary Figures 2C, D**).

Next, DEGs from the four scallop families were compared by Venn diagrams, separately from infected and uninfected conditions (**Figure 4C**). In the uninfected state, we observed 149 DEGs exclusive to the resistant F10 and F12 scallop families, while 100 DEGs were exclusive to the susceptible F8 and F9 families. Only 7 DEGs were common to the four families (**Figure 4C**). After infection, only 34 DEGs were found as exclusive to the resistant F10 and F12 scallop families, while 106 DEGs were exclusive to the susceptible F8 and F9 families. After infection, no common DEGs were found between the four families (**Figure 4C**).

We validated our RNA-seq results by quantifying the expression of 10 specific DEGs found between resistant with respect to susceptible larvae by RT-qPCR (**Supplementary Table 3**; **Figure 3**). Transcripts encoding a Mucin (*muc*), RIG-like receptor (*ddx58*), a myeloperoxidase (*MPO*), interleukin-17 like receptor (*IL17R*), Toll-like receptor (*TLR*), Caveolin, Cadherin 23, Tyrosine Kinase Fyn, Neuronal acetylcholine receptor subunit alpha-10 (*CHRNA10*), and a Copper Zinc Superoxide dismutase (*CuZnSOD*) were analyzed. The fold change values were consistent between methods, as supported by correlation analysis (uninfected, R^2^: 0.9320; infected, R^2^: 0.9352).

### 3.4 Infected resistant larvae overexpress immune-related functions and underexpress DNA and cell division processes

**Figure 5:**
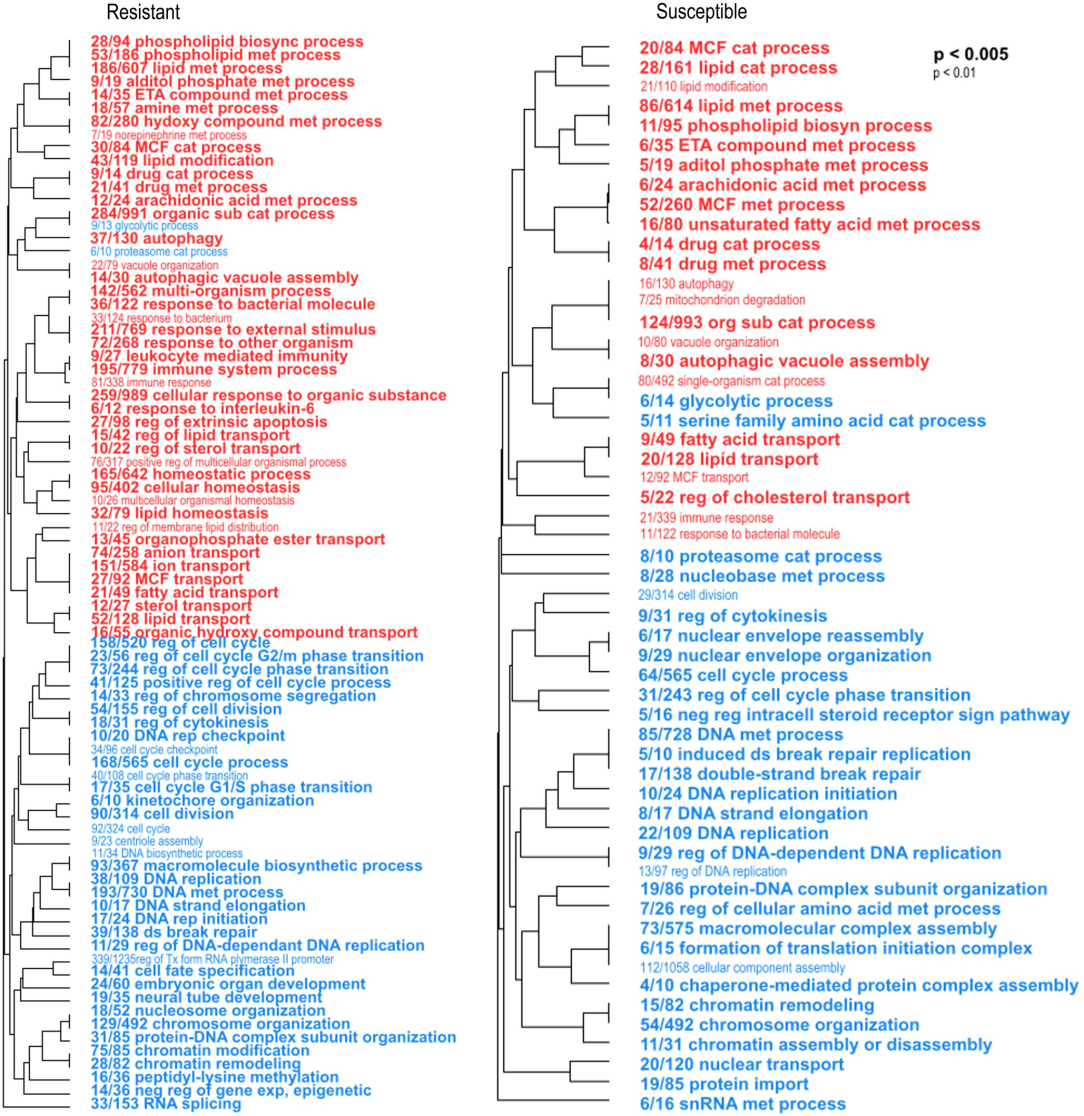
Enriched biological processes occurring in resistant and susceptible *Argopecten purpuratus* scallop larvae in response to *Vibrio bivalvicida* VPAP30. Hierarchical clustering trees of enriched biological processes (BP) in resistant (left panel) and susceptible (right panel) infected larvae by RBGOA. Red terms indicate an over-representation of the BP and blue terms indicate an underrepresentation of the BP in infected larvae. met: metabolic, cat: catabolic; sub: substance, reg: regulation, rep: replication, neg: negative, exp: expression. The fraction preceding each BP term indicates the number of genes annotated within the term from the total number of genes related to this BP that pass an unadjusted p-value threshold.

Then, we determined the biological processes enriched in each larval phenotype in response to the pathogen by comparing the transcriptomes of infected versus uninfected larvae. To do this, we analyzed the response of resistant and susceptible larvae to *V. bivalvicida* VPAP30 separately by RBGOA, using DEGs found between before (0 hpi) and after (8 hpi) infection (**Figure 5**, **Supplementary Table 6**). Enriched immune-related biological processes were more abundant (numerous) in resistant larvae 8 h hpi. Indeed, we found a total of nine overexpressed immune categories in resistant larvae, which included immune response, immune system process, cell-mediated immunity, and response to interleukin; response to bacterium and bacterial molecule; to external stimulus and to other organisms. In susceptible larvae we found only two overexpressed immune categories, namely immune response, and response to molecule of bacterial origin (**Figure 5**). Overexpressed immune genes in infected resistant larvae encoded several Toll-like receptors (log_2_FC 0.7, 1.2), a C-type lectin (log_2_FC 0.89), TNF and NF-κB immune signaling (log_2_FC 0.38, 1.3) as well as the antimicrobial peptide Big defensin *ApBD1* (log_2_FC 2.37) (**Supplementary Table 6 tab 1**). Remarkably, none of these genes were found in the immune categories enriched in infected susceptible larvae (**Supplementary Table 6 tab 2**). A strong underrepresentation of non-immune biological processes was also observed in resistant larvae. Biological processes related to DNA replication and metabolism, chromatin remodeling, and cell division contained mostly downregulated genes in both larval phenotypes after infection (**Figure 5**; **Supplementary Table 6**). But resistant larvae showed a higher number of these biological processes and a higher number of downregulated genes within each of them (**Figure 5**; **Supplementary Table 6 tab 1**). It is worth mentioning that many processes associated with lipid metabolism were enriched in both larval phenotypes of resistance, but a greater number were observed in the resistant scallop larvae.

### 3.5 Immune-related biological processes are overexpressed in uninfected resistant larvae

**Figure 6:**
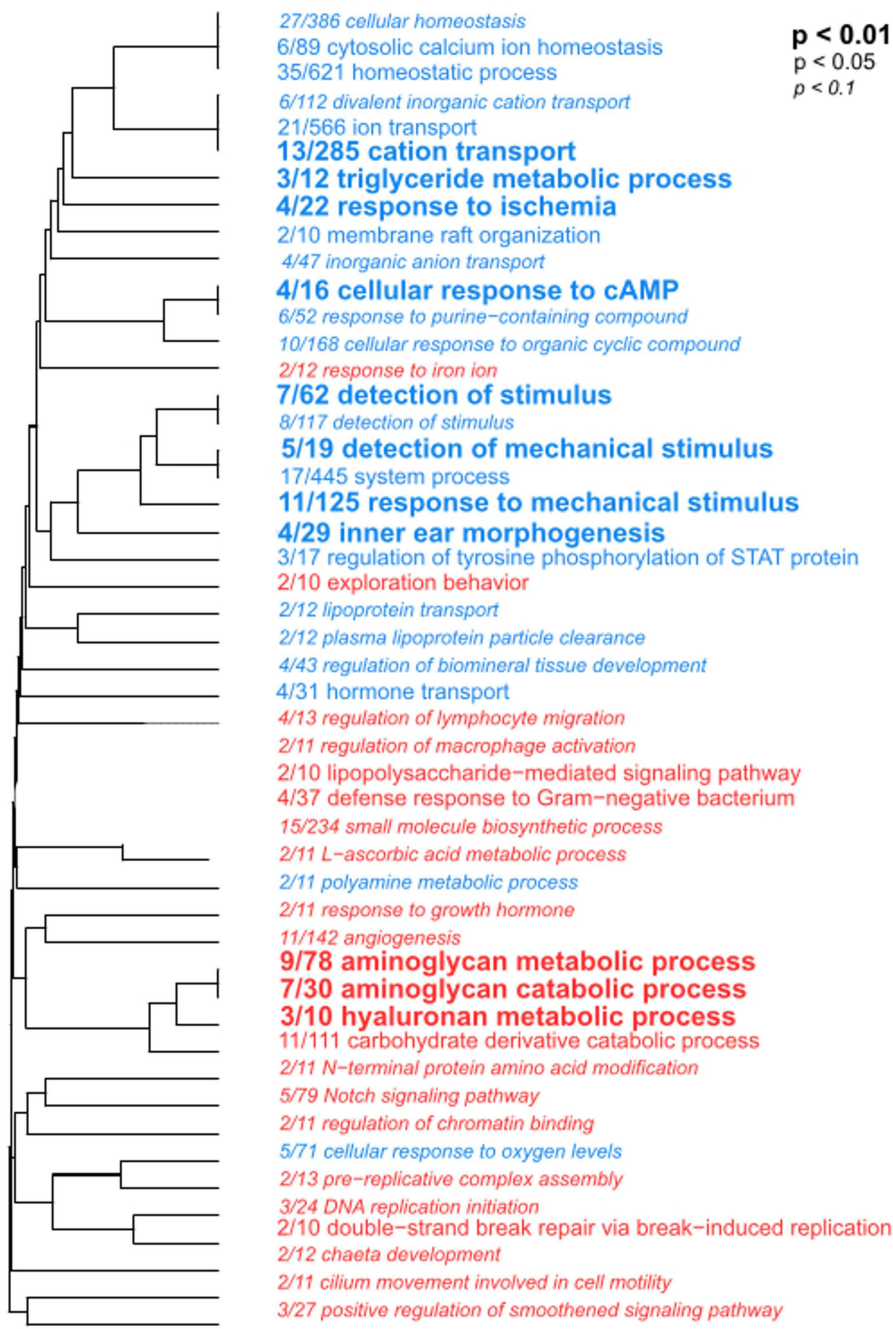
Functional enrichment analysis with DEGs found between uninfected resistant and susceptible *Argopecten purpuratus* scallop larvae. Hierarchical clustering tree of biological processes (BP) showed significantly enriched categories in the resistant (terms in red) compared to susceptible (terms in blue) uninfected larvae by RGBOA. The fraction preceding each BP term indicates the number of genes annotated within the term from the total number of genes related to this BP that pass an unadjusted p-value threshold Letter size indicates different p-values.

We performed a functional enrichment analysis using the DEGs found between uninfected resistant and susceptible larvae (0 hpi), using a Rank-Based Gene Ontology Analysis with adaptive clustering (RBGOA). By this analysis, we identified enriched biological processes containing genes that are either over or under expressed in the resistant compared to the susceptible larvae. The transcriptome of uninfected resistant larvae displayed several enriched immune-related biological processes, such as lipopolysaccharide-mediated signaling pathway, defense response to Gram-negative bacterium, and regulation of immune cell migration and activation. These enriched immune-related biological processes mostly contained overexpressed genes involved in immune recognition and antimicrobial activity, such as lipopolysaccharide-binding proteins (*lbp.* log_2_FC 0.84), lysozyme (*lys*, log_2_FC 1.02), and bactericidal permeability-increasing protein (*bpi*, log_2_FC 0.54) (**Figure 6**; **Supplementary Table 7 tab 1**). Besides, two of the most enriched biological processes were associated with aminoglycan metabolic and catabolic processes, with genes involved in chitin remodeling such as N-acetylglucosamine-6-sulfatase and chitinase overexpressed in resistant larvae. Categories such as morphogenesis, biomineral tissue development, and angiogenesis were underexpressed in uninfected resistant larvae (**Figure 6**; **Supplementary Table 7 tab 1**).

### 3.6 Immune-related biological processes discriminate resistant and susceptible infected larvae

To determine the biological processes differentiating the larval phenotypes in response to the pathogen, we focused on DEGs between resistant and susceptible larvae after infection (**Supplementary Table 3**; **Supplementary Figure 3**). Several enriched biological processes related to the recognition, signaling pathways and effectors of the immune response were highly enriched with DEGs from resistant infected larvae. Some of these biological processes were related to response to abiotic stimulus, positive regulation of different inflammatory cytokines, immune cell activation and iron homeostasis (nutritional immunity) (**Supplementary Table 7 tab 2**). In contrast, categories such as response to increased oxygen levels, membrane raft organization, ion, water, and lipid transport, as well as regulation of wound healing were enriched by under expressed genes, with caveolins found associated with all those biological processes.

### 3.7 Frontloaded expression of immune genes clusters according to larval resistance phenotype

**Figure 7:**
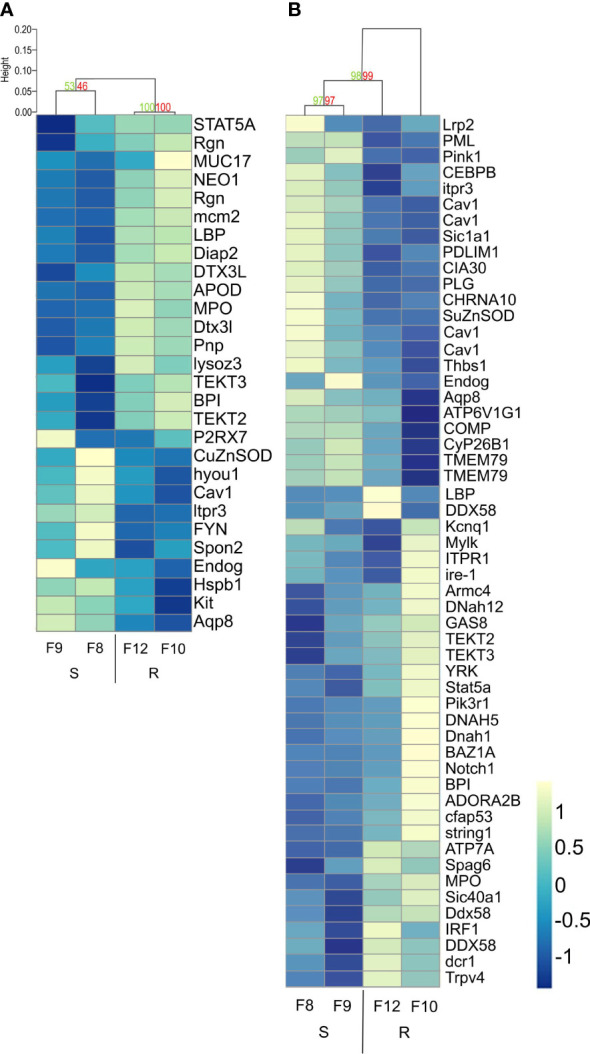
Hierarchical clustering heatmap from differentially expressed immune-related genes found between resistant and susceptible *Argopecten purpuratus* scallop larvae. DEGs were obtained from the immune-related biological processes enriched by the RBGOA analysis. **(A)**. uninfected scallop larvae. **(B)**. infected scallop larvae. AU (Approximately Unbiased) p-value and BP (Bootstrap Probability) value are shown in red and green, respectively for each cluster. Expression log_2_ fold change values are represented through the color scale from blue (relative low level of gene expression) to yellow (relative high level of gene expression). Gene symbols can be found in the online HUGO Gene Nomenclature Committee database and in **Supplementary Table 8**.

Finally, we compared the differential expression of the immune genes from the enriched immune-related biological processes by hierarchical cluster analysis with p-values, to assess clustering uncertainty (**Figure 7**; **Supplementary Table 8**). In uninfected larvae, differentially expressed immune genes clustered according to host phenotype, but the two resistant families (F10 and F12) showed stronger clustering than the two susceptible families (F8 and F9) (**Figure 7**). Recognition molecules such as *lbp* (log_2_FC 0.84), immune signaling pathway related proteins and antimicrobial effectors including a *bpi* (log_2_FC 0.54), a *lys* (log_2_FC 1.02), and a myeloperoxidase (*MPO*, log_2_FC 0.95) were overexpressed in uninfected resistant larvae (**Figure 7A**; **Supplementary Table 8 tab 1**).

In response to *Vibrio* infection, the gene expression was more variable within each larval family, and the clusters were less supported by the data compared to before infection. Still, we identified discrete clusters of overexpressed genes in infected resistant larvae. These clusters included overexpressed genes related to phagocytosis and intracellular killing, which appears as one function discriminating host phenotypes (**Figure 7B**). Overexpressed genes in resistant larvae included pattern recognition receptors (*RLRs* such as *DDX58* and *LBPs*, log_2_FC 0.49-1.6), copper transporter (*ATP7A*, log_2_FC 0.36), myeloperoxidase (*MPO*, log_2_FC 0.95) and *TRPV4* (involved in LPS-induced macrophage phagocytosis, log_2_FC: 1.16) (**Figure 7B**; **Supplementary Table 8 tab 2**). The difference in the expression of these immune genes was low to moderate, showing a log_2_FC range between 0.54 and 1.02 from uninfected and between 0.36 and 1.6 from infected resistant larvae (**Supplementary Table 8**).

## 4 Discussion

In the present study, we showed that the resistance of scallop larvae to *Vibrio bivalvicida* VPAP30 is associated with the overexpression of immune biological processes in both uninfected and infected larvae. This suggests that the immune transcriptional frontloading and the enhanced antimicrobial response of resistant larvae play an essential role in controlling pathogenic infection by this vibrio. In addition, the *Vibrio* transcriptional response does not vary significantly with host resistance or susceptibility, indicating that host factors may determine the infection outcome by inhibiting the bacterial invasion strategy.

Transcriptional frontloading, defined has the higher constitutive expression of certain genes, has been proposed as an important mechanism promoting species resilience under changing conditions (29). Here, uninfected resistant scallop larvae overexpressed immune genes involved in LPS recognition (LBPs) and in the production of antibacterial effectors (BPI, lysozyme, MPO) (30), suggesting a frontloaded antibacterial immune status. In oyster families resistant to POMS, frontloaded immune genes were related to immune signaling pathways (17). In other marine invertebrates such as arthropods and corals, the transcriptional frontloading has been shown to contribute to tolerance and cross‐tolerance between environmental stressors such as heat and/or hypoxia (31, 32). Here, a higher basal expression levels of key immune genes could prepare resistant scallop larvae to rapidly respond and inhibit the *Vibrio* infection.

In multicellular organisms, the phagocytic process by immune cells plays an essential role in the control of microbial diseases (33). In bivalves, hemocytes are the only mobile immunocompetent cells that display intracellular killing activities mediated by phagolysosome formation and reactive oxygen species (30). Results obtained here suggest that the success of scallop larvae to resist infection relies to some extent upon the immunocompetence of hemocytes. Compared to its susceptible counterpart, infected resistant larvae expressed Copper-transporting ATPase *ATP7A*, which gene product is a key regulator of copper homeostasis involved in macrophage bactericidal activity and nutritional immunity in vertebrates (34). It was earlier shown that copper homeostasis is a key determinant of *Vibrio* infections in bivalves (35, 36). They also expressed myeloperoxidase (*MPO*), which gene product is a major constituent of neutrophils that combined with hydrogen peroxide and chloride represents a potent oxidative killing antimicrobial system (37). Another overexpressed gene was the transient receptor potential vanilloid 4 (*TRPV4*), an ion channel involved in LPS-induced macrophage phagocytosis (38). The expression of phagocyte-associated immune genes in resistant scallop larvae highlights the relevance of hemocyte’s enhanced intracellular killing of microbial pathogens. Globally, results showed that differentially expressed immune biological processes and immune gene expression allow discriminating resistant and susceptible larvae from both uninfected and infected conditions.

Bivalve hemocytes also produce and secrete antimicrobial proteins and peptides (AMPs). Previous work showed that *A. purpuratus* hemocytes produce the AMPs BPI and Big defensin ApBD1, which regulate the scallop microbiota (9). We showed here that infected resistant scallop larvae overexpressed BPI and ApBD1 while no significant differential expression was found in infected susceptible larvae. This result agrees with previous findings on resistant oysters to POMS, where the expression of Big Defensins was induced only in resistant oyster families during infection (39). Overall, the higher expression of AMPs in resistant scallop larvae both before infection and during response appears as a key determinant on the outcome of the infection and in scallop-microbe interactions.

Immune defense is energetically costly, and an effective response involves the reallocation of energy from metabolism toward the immune system (40). For instance, innate immune signaling in *Drosophila* shifts lipid metabolism from triglyceride storage to phospholipid synthesis to support the immune response (41). Recently, it has been shown that the morphological changes associated with the development affected the energy metabolism of *A. purpuratus* larvae, and that veliger larvae exhibit the lowest metabolic capacity to overcome a bacterial challenge (19). We showed in the present work that resistant scallop larvae have a higher expression of immune genes both at basal level and during early infection. Furthermore, after exposure to the pathogen there is a strong activation of many processes associated with lipid metabolism, suggesting the mobilization of these energy molecules to support the immune response (including some polyunsaturated fatty acids). This activation is stronger (and with many more genes annotated) in the resistant than in the susceptible larval phenotype. This would indicate a greater energetic capacity to support the immune response in the resistant than in the susceptible larvae, which also express much fewer biological processes associated with immune response than in the resistant after infection. These biological processes suggest that the reallocation of energy from scallop larval development to immunity contributes to the resistance phenotype. The impact of the metabolic cost on the other traits of interest such as growth should be considered if selective breeding is attempted (13). In the case of scallop, the selection of resistant larvae can be considered as an opportunity, since they would have a better energetic efficiency to resolve the infection.

Vibrios are commonly associated with mass mortalities in larvae, juveniles, and adults of several bivalves such as oysters, mussels, clams, and scallops (42). Here, we identified some of the early adaptations that *V. bivalvicida* undergo during the colonization process of scallop larvae trough a natural route of infection, such as chemotaxis and carbohydrate metabolism. The activation of a chemotactic response is expected to be due to the presence of the host-derived chemoattractant polymer chitin, as shown in the *Vibrio fisheri* - squid association (43). A reprograming of the carbohydrate metabolism has also been observed in *V. tasmaniensis* and *V. crassostreae* infecting adult oysters (28), and *V. vulnificus* infecting humans (44), which suggests a conserved adaptation to this energetically demanding process.

Regarding virulence, we identified an UDP-N-acetylglucosamine C-6 dehydrogenase with 100% identity with the *TviB* protein from *Salmonella enterica* serovar Typhi. This protein is necessary for the synthesis of the VI capsular polysaccharide, which has so far not been identified in vibrios. In humans, this capsular polysaccharide contributes to the pathogenesis of typhoid fever, mediating the evasion of neutrophil phagocytosis while promoting macrophage phagocytosis (45). Moreover, in the oyster-vibrio model of interaction it has been demonstrated that the LPS structure can impact recognition, virulence, and environmental survival (46). Therefore, the role of polysaccharide synthesis as a survival/virulence strategy in scallop larvae should be explored in *V. bivalvicida.* We also highlighted the differential expression of components belonging to type II and III secretion systems and one thermolabile hemolysin (*tlh)*. The activity of type VI secretion systems has been functionally validated on its contribution to virulence in the oyster model (28, 47) and the secretion of thermo-resistant toxins has a significant role in *V. bivalvicida* virulence toward scallop larvae (4). The present work introduces mechanisms to further study on its contribution to the infection success of larvae by vibrios. Namely, the chemoattractant effect of host-derived chitin for host encounter, the polysaccharide synthesis as a possible immune evasion strategy, and the role of the secretion of toxic effectors in the already reported cilial damage and intestinal colonization by *V. bivalvicida.* Overall, we identified candidate virulence factors among differentially expressed *Vibrio* genes that span through a general infective process, which includes chemotaxis, immune evasion, and the production and secretion of toxic effectors. However, at this early time point of infection (8 hpi) the overall bacterial strategy does not discriminate between the resistant or susceptible larvae.

This study opens the way to future investigations to characterize the key host mechanisms determining the infection outcome. Our data suggest that an early immune response is essential. It will be key to identify at which point in the colonization process *V. bivalvicida* is effectively controlled by the resistant scallop larvae. Considering the crucial role that hemocytes appear to play in scallop resistance, future functional studies should focus on the oxidative response, copper homeostasis, and AMP expression in the control of *Vibrio*. On the pathogen side, it will also be important to test the cytotoxic activity of *V. bivalvicida* towards the hemocytes, a conserved mechanism found in *Vibrio* pathogenic for oysters (28) and if evidenced, determine which of the putative virulence factors identified here are involved.

Finally, the present study showed that the scallop resistance to a pathogenic *Vibrio* is related to host genetics. Indeed, contrasted phenotypes represented by two families each, resulted to be half-siblings, highlighting the strong genetic basis in the resistance of scallop larvae to *V. bivalvicida*. We cannot exclude the possibility that high allelic variation, gene copy number variation (CNV) or presence/absence variation (PAV) affecting immune genes could contribute to the differential gene expression detected between scallop families, and these factors need to be taken into account if breeding is considered. Selective breeding with inbreeding control has been proposed as the top strategy for genetic improvement of mollusks, where growth rate and disease resistance can be improved by 10% and 15% per generation, respectively, by implementing individual or family-based selection (13). By contributing important knowledge on the molecular bases of the resistance of scallop larvae, this study provides key host gene candidates to detect associated SNPs to be tested for marker-assisted selective breeding.

## Data availability statement

The datasets presented in this study can be found in online repositories. The names of the repository/repositories and accession number(s) can be found below: https://www.ncbi.nlm.nih.gov/, PRJNA891447.

## Author contributions

PS, DD-G, and KB conceived and planned the experiments. DO, RR, GL, AM, and KM carried out the experiments. EJ and RF planned and carried out the bioinformatic analyzes. EJ, DO, RR, GL, AM, and KM contributed to sample preparation. EJ, KB, PS, DO, RR, and DD-G contributed to the interpretation of the results. All authors provided critical feedback and helped shape the research, analysis, and manuscript. All authors contributed to the article and approved the submitted version.
