# Supplementary material for: Resistance of Argopecten purpuratus scallop larvae to vibriosis is associated with the front-loading of immune genes and enhanced antimicrobial response
Source: Front Immunol. 2023 Mar 3;14:1150280. doi: 10.3389/fimmu.2023.1150280 (PMC10020363; doi:10.3389/fimmu.2023.1150280)
Supplement: Supplementary file 1 [file Table_3.docx]

Supplementary Material

**Resistance of *Argopecten purpuratus* scallop larvae to vibriosis is associated with the front-loading of immune genes and enhanced antimicrobial response**

**Eduardo Jeria^1†^, Daniel Oyanedel^1†^, Rodrigo Rojas^2^, Rodolfo Farlora^3,4^, German Lira^5^, Ana Mercado^5^, Katherine Muñoz^1^, Delphine Destoumieux-Garzón^6^, Katherina Brokordt^5^ & Paulina Schmitt^1*^**

***Correspondence**

Corresponding Author

[paulina.schmitt@pucv.cl](mailto:paulina.schmitt@pucv.cl)

^†^ These authors contributed equally to this work and share first authorship

## Supplementary Figures


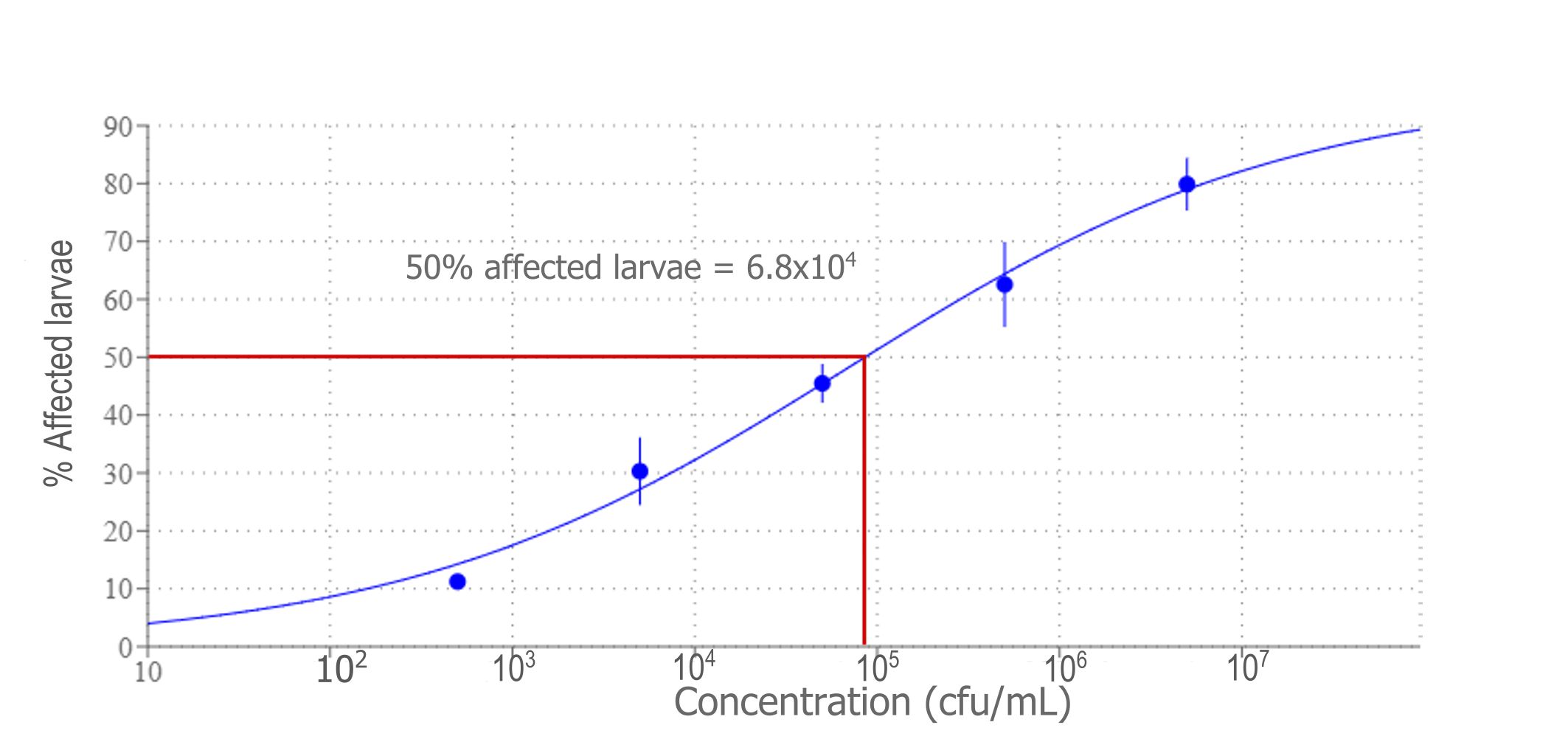


**Supplementary Figure 1. Determination of *Vibrio bivalvicida* VPAP30 dose to affect 50% of scallop larvae of multiparental origin in 24 h.** 100 larvae/well were placed in a final volume of 4 mL of SSW in 6-well plates with gentle aeration. The VPAP30 strain was then added and tested at different concentrations (1x10^6^, 1x10^5^, 1x10^4^, ^1x^10^3^ and 1x10^2^ cfu/mL). As a control, 6 wells with pathogen-free larvae were included. Larvae were incubated at 18°C and 24 h after exposure the larval status was assessed as previously described (Rojas et al. 2019), using an Olympus CKX41 inverted optical microscope (200X). Clinical signs of larval infection, such as no ciliary movement, erratic swimming, larval crowding, veil shedding and bacterial swarming around larvae were assessed. Larvae that showed clinical signs were considered as affected by the pathogen. After visual analysis, larvae were stained with Lugol to determine the total number of larvae and the percentage of affected larvae. The percentage of affected larvae was calculated using the formula: % affected larvae =100* (Number of affected larvae)/(Number of total larvae).

**
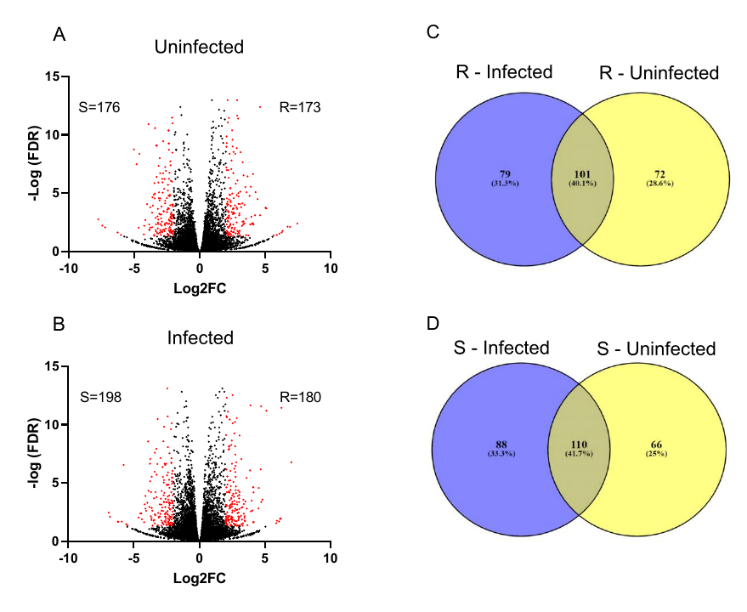
**

**Supplementary Figure 2.** **Volcano plots (panels A-B) from significant differentially expressed genes (DEGs) determined from pair-wise comparisons** between A. uninfected resistant (R) vs. susceptible (S) scallop larvae, B. infected resistant (R) vs. susceptible (S) scallop larvae transcriptomes. Statistically significant transcripts are shown by |log2 fold change| ˃ 2 vs -log10 (p-value) in red. **Panels C- D.** Venn diagrams of unique and common transcripts among the transcriptomes from uninfected and infected resistant (C) and susceptible (D) scallop larvae, showing the number and percentage of all classified transcripts included for each category.


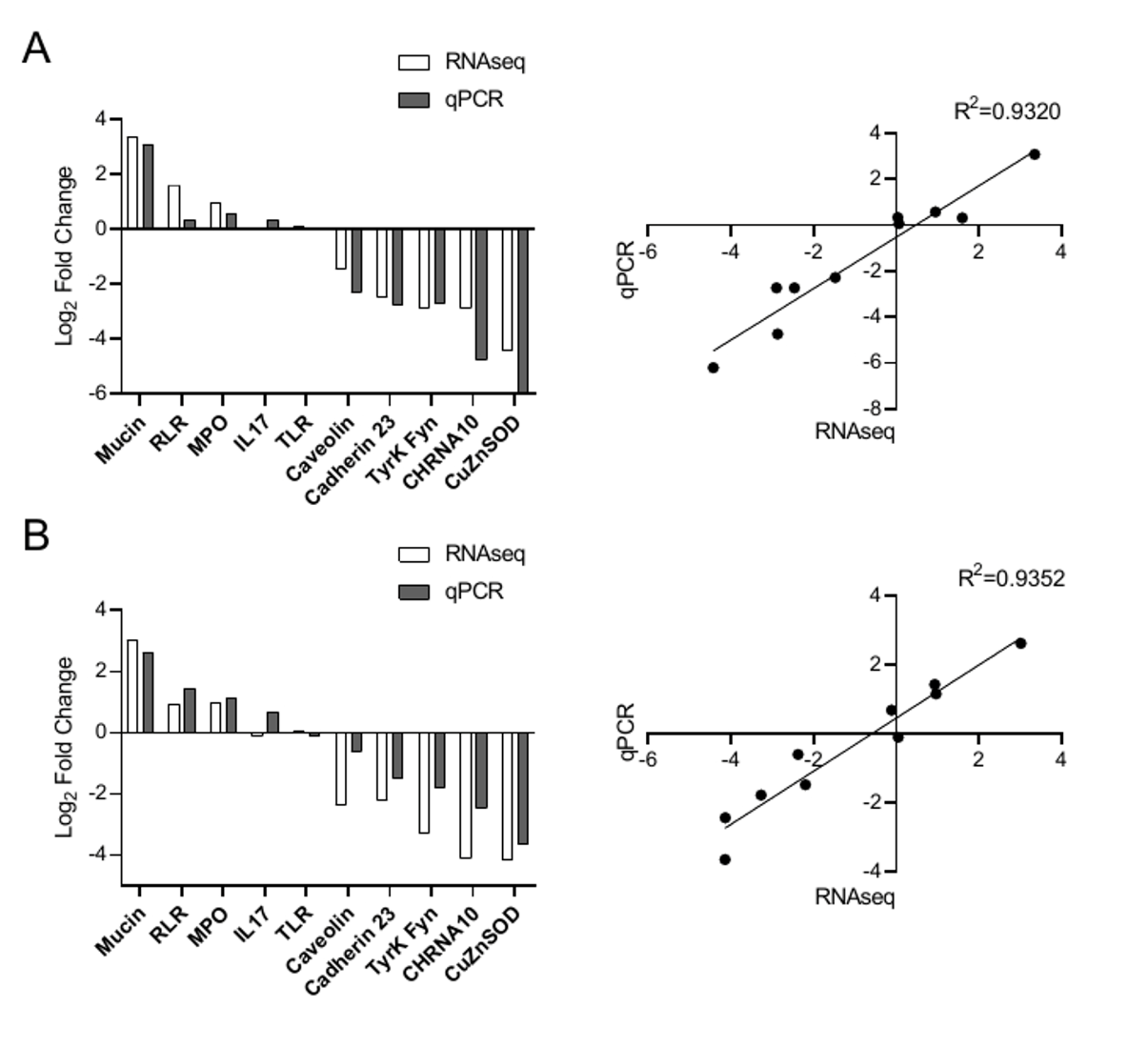


**Supplementary Figure 3. RT-qPCR validation of RNA-seq results from uninfected (A) and infected (B) resistant with respect to susceptible larvae under the same conditions.** The same RNA used for the construction of cDNA libraries was used for RT-qPCR amplification. Relative expressions of immune related genes were obtained according to the 2^−ΔΔCq^ method using the geometric mean of the measured quantitation cycle (Cq) values of the constitutively expressed genes elongation factor 1 α (EF-1α) (Muñoz et al 2019), and the enzyme Glyceraldehyde 3-phosphate dehydrogenase GAPDH to normalize the measured Cq values of the target genes. Results are expressed as mean values ±SD from 3 pools of 15,000 larvae per experimental condition. Correlation analysis between RNA-seq and RT-qPCR log2 fold change results from pairwise comparisons between the log2fold change values from both approaches. R2: Spearman Correlation coefficient.


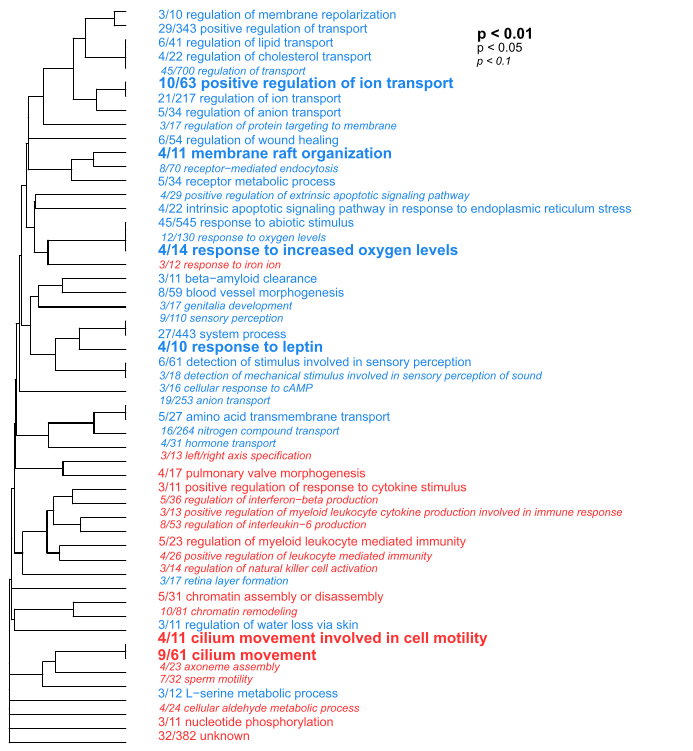


**Supplementary Figure 4. Functional enrichment analysis with DEGs found between resistant with respect to susceptible scallop larvae infected with *Vibrio bivalvicida* VPAP30 for 8 h.** Hierarchical clustering tree of biological processes (BP) showed significantly enriched categories in the resistant compared to susceptible infected larvae by RGBOA. Red terms indicate an over-representation of the BP and blue terms indicate an underrepresentation of the BP in the infected resistant scallop larvae. The size of the terms is associated with the P value. The fraction preceding each BP term indicates the number of genes annotated within the term from the total number of genes related to this BP that pass an unadjusted p-value threshold.
